# iCardio e Ferramentas Baseadas em *Business Intelligence*: Oportunidades na Saúde Pública

**DOI:** 10.36660/abc.20260419

**Published:** 2026-07-14

**Authors:** Fernando Yue Cesena

**Affiliations:** 1 Instituto Dante Pazzanese de Cardiologia São Paulo SP Brasil Instituto Dante Pazzanese de Cardiologia, São Paulo, SP – Brasil

**Keywords:** Saúde Pública, Doenças Cardiovasculares, Gestão da Informação

Nas últimas décadas, temos testemunhado uma explosão na geração de dados em todos os campos. Na área da saúde, o uso generalizado de prontuários médicos eletrônicos e o advento de dispositivos vestíveis de monitoramento, por exemplo, têm contribuído para a geração de enormes volumes de dados de saúde. Na saúde pública, agora é mais fácil do que nunca usar métodos computacionais para criar conjuntos de dados com informações sobre morbidade e mortalidade. Um dos desafios é que os métodos de coleta de dados e as características da população, como a pirâmide etária, variam entre países e ao longo do tempo, dificultando a comparação e a análise de tendências temporais. Essa é a tarefa do estudo Carga Global de Doenças (*Global Burden of Disease*, https://www.healthdata.org/research-analysis/gbd), que padroniza indicadores de mais de 400 desfechos de saúde e fatores de risco em mais de 200 países e territórios, permitindo comparações justas entre países e tendências em dados epidemiológicos ao longo do tempo, inclusive no Brasil.^1,2^ Outro desafio é que a informação muitas vezes está fragmentada em conjuntos de dados distintos. Essa é a tarefa da chamada *Business Intelligence*, que pode ser usada para criar ferramentas que transformam dados brutos e fragmentados, frequentemente coletados de diferentes fontes, em percepções visuais, muitas vezes dinâmicas e interativas, que permitem atualizações em tempo real, facilitando a interpretação de dados como um todo e apoiando o processo de tomada de decisão.^3^

Nesta edição dos Arquivos Brasileiros de Cardiologia, Souza et al.^4^ relatam um estudo que utiliza o iCardio, uma plataforma online que aproveita a *Business Intelligence* para visualizar dados epidemiológicos em cardiologia intervencionista e cirurgia cardíaca do DATASUS no Brasil (www.iats.com.br/icardio).^4^ O iCardio, que faz parte de um projeto financiado pelo Conselho Nacional de Desenvolvimento Científico e Tecnológico (CNPq), foi desenvolvido com dados do Sistema de Informações Hospitalares (SIH) e do Sistema de Informação sobre Mortalidade (SIM). Os indicadores incluem o número de procedimentos e internações de alta complexidade, o número de procedimentos por habitante, a localização geográfica onde o atendimento médico foi prestado, o deslocamento médio até o serviço de saúde, o tempo de internação, a taxa de reinternação, as taxas de mortalidade hospitalar e de mortalidade em até 30 dias após o procedimento ou alta hospitalar, e os custos. É importante ressaltar que os dados podem ser estratificados por sexo, faixa etária, raça/cor da pele, localização, tipo de atendimento (urgente/eletivo), diagnóstico e procedimento.^4,5^

Os autores relatam um exemplo de uso do iCardio, demonstrando disparidades regionais nos procedimentos cardiovasculares de alta complexidade no Brasil. Eles constataram uma taxa bruta de mortalidade mais alta na região Norte e uma taxa de mortalidade mais baixa na região Sudeste. A região Sul apresentou a maior taxa padronizada de procedimentos, enquanto a região Nordeste apresentou o maior deslocamento médio para atendimento médico.^4^

O iCardio ilustra como podemos aproveitar novas tecnologias para criar ferramentas que facilitem a integração de informações relevantes, gerem percepções e incentivem ações por parte das autoridades de saúde. No entanto, o uso de ferramentas de *Business Intelligence* requer algumas considerações. Primeiramente, pode-se argumentar que a qualidade dos dados seja o aspecto mais importante. O famoso ditado "*garbage in, garbage out*" ("lixo entra, lixo sai") se aplica perfeitamente aqui. Se a ferramenta for alimentada com dados "sujos", os resultados serão tendenciosos ou errôneos, e as consequências podem ser catastróficas. Portanto, a vigilância contínua e os esforços para melhorar a qualidade dos dados, reduzindo a subnotificação e as inconsistências, são imprescindíveis. Em segundo lugar, idealmente, a ferramenta deve ser abrangente e inclusiva para alcançar todo o país. Seria excelente se uma ferramenta como o iCardio incluísse não apenas indivíduos do Sistema Único de Saúde (SUS), mas também aqueles com planos de saúde e do setor privado. Isso permitiria análises verdadeiramente de âmbito nacional. Em terceiro lugar, seria ótimo se esse tipo de ferramenta incluísse dados adicionais, como morbidades não cardiovasculares, uso de medicamentos, hábitos de vida, fatores ambientais e socioeconômicos. Em quarto lugar, projetos como este devem ter continuidade, de preferência com atualizações anuais, para manter sua razão de existir. Em quinto lugar, ferramentas como essa devem desencadear ações, estimulando discussões sérias que levem à proposição de planos ou apoiem o processo de tomada de decisão. Em sexto lugar, é necessário monitorar a eficácia dessas ações para fornecer uma retroalimentação que possa aprimorar o processo como um todo.

Segundo a OCDE (Organização para a Cooperação e Desenvolvimento Econômico), o Brasil coleta uma grande quantidade de dados digitais de saúde, mas está atrás de outros países em relação à disponibilidade, divulgação, governança e integração de dados.^6^ O iCardio é um exemplo claro de como podemos preencher essa lacuna.

Em conclusão, os autores do artigo, bem como todos os envolvidos no desenvolvimento do iCardio, devem ser parabenizados. Esperamos que esta ferramenta não só gere artigos científicos com belas tabelas e figuras, mas, sobretudo, que contribua para orientar as políticas de saúde pública. O objetivo final é utilizar este tipo de ferramenta para melhorar a qualidade da assistência, otimizar a alocação de recursos, prevenir eventos adversos e reduzir as disparidades na utilização dos serviços de saúde e nos desfechos.
