# Supplementary material for: iCardio e Ferramentas Baseadas em Business Intelligence: Oportunidades na Saúde Pública
Source: Arq Bras Cardiol. 2026 Jul 14;123(4):e20260419. [Article in Portuguese] doi: 10.36660/abc.20260419 (PMC13398895; doi:10.36660/abc.20260419)
Supplement: Translation

# Translation: iCardio And Business Intelligence-Based Tools: Opportunities in Public Health

**DOI:** 10.36660/abc.20260419i

**Authors:** Fernando Yue Cesena

**Affiliations:** 1 São Paulo SP Brazil Instituto Dante Pazzanese de Cardiologia, São Paulo, SP – Brazil

**Keywords:** Public Health, Cardiovascular Diseases, Information Management

We have witnessed an explosion of data generation in every field over the last decades. In healthcare, the widespread use of electronic medical records and the advent of wearable monitoring devices, for instance, have contributed to the generation of huge volumes of health data. In public health, it is now easier than before to use computational methods to create datasets with morbidity and mortality data. One challenge is that data collection methods and population characteristics, such as the age pyramid, vary across countries and over time, making it difficult to compare and analyze temporal trends. This is the task of the Global Burden of Disease (https://www.healthdata.org/research-analysis/gbd), which standardizes indicators of more than 400 health outcomes and risk factors in more than 200 countries and territories, allowing fair comparisons between countries and trends in epidemiological data over time, including in Brazil.^1,2^ Another challenge is that information is often fragmented in distinct datasets. This is the task of the so-called Business Intelligence, which may be used to create tools that transform fragmented, raw data frequently collected from different sources into visual insights, often dynamic, interactive, that allow real-time updates, facilitating whole data interpretation and supporting the decision-making process.^3^

In this issue of *Arquivos Brasileiros de Cardiologia*, Souza et al.^4^ report a study using iCardio, an online platform that leverages Business Intelligence to visualize epidemiological data in interventional cardiology and heart surgery from DATASUS in Brazil (www.iats.com.br/icardio).^4^ iCardio, which is part of a project sponsored by the National Council for Scientific and Technological Development (Portuguese: *Conselho Nacional de Desenvolvimento Científico e Tecnológico* [CNPq]), was developed with data from the Hospital Information System (Portuguese: *Sistema de Informações Hospitalares* [SIH]) and Information System of Mortality (Portuguese: *Sistema de Informação sobre Mortalidade* [SIM]). The indicators include the number of high-complexity procedures and hospitalizations, the number of procedures per inhabitants, the geographical location where medical care was provided, the average displacement to the healthcare facility, the hospitalization length of stay, the re-hospitalization rate, the rates of in-hospital mortality and mortality up to 30 days after the procedure or hospital discharge, and costs. Importantly, data may be stratified per sex, age range, race/skin color, location, type of care (urgent/elective), diagnosis, and procedure.^4,5^

The authors report an example of iCardio's use, demonstrating regional disparities in high-complexity cardiovascular procedures in Brazil. They found a higher crude mortality rate in the North region and a lower mortality rate in the Southeast region. The South region had the highest standardized rate of procedures, while the Northeast had the highest average displacement to medical care.^4^

iCardio illustrates how we can leverage new technologies to create tools that enable the easy integration of relevant information, generate insights, and trigger actions by health authorities. However, the use of Business Intelligence tools warrants consideration. First, data quality is arguably the most important aspect. The famous "garbage in, garbage out" perfectly applies here. If the tool is fed with "dirty" data, the results will be biased or erroneous, and the consequences can be catastrophic. Therefore, continuous vigilance and attempts to improve data quality, reducing undernotification and inconsistencies, are mandatory. Second, ideally, the tool must be comprehensive and inclusive to reach the whole country. It would be superb if a tool like iCardio included not only individuals from the Brazilian Unified Health System (Portuguese: *Sistema Único de Saúde* [SUS]) but also those with health insurance and in the private sector. This would allow real nationwide analyses. Third, it would also be great if this kind of tool included additional data, such as non-cardiovascular morbidities, medication use, lifestyle habits, environmental factors, and socioeconomic factors. Fourth, projects like this must have continuity, preferably with annual updates, to keep their rationale for existing. Fifth, tools like this must be actionable, sparking serious discussions that lead to the proposal of plans or support the decision-making process. Sixth, it is necessary to monitor the effectiveness of these actions to provide feedback that can improve the overall process.

According to the OECD (Organisation for Economic Co-operation and Development), Brazil collects a large amount of digital health data, but is behind other countries regarding data availability, reporting, governance, and integration.^6^ iCardio is a clear example of how we can fill this gap.

In conclusion, the authors of the article, as well as those involved in developing iCardio, should be congratulated. Hopefully, this tool will generate not only scientific articles with beautiful tables and figures, but, most importantly, help to guide public health policies. The ultimate goal is to leverage this kind of tool to improve the quality of care, optimize resource allocation, prevent adverse events, and reduce disparities in healthcare utilization and outcomes.
